# Conservative Management of Horizontal Fracture and Lateral Luxation in the Anterior Region: A 20-Year Follow-Up Case Report With Late Complications

**DOI:** 10.1155/crid/8898427

**Published:** 2025-08-06

**Authors:** José Espona, Fernando Duran-Sindreu, Miguel Roig, Elena Roig

**Affiliations:** ^1^Department of Restorative Dentistry, International University of Catalonia, Barcelona, Spain; ^2^Department of Endodontics, International University of Catalonia, Barcelona, Spain; ^3^Department of Orthodontics, International University of Catalonia, Barcelona, Spain

**Keywords:** dental implantation, tooth fractures, tooth loss, tooth luxation

## Abstract

The protocolization of dental trauma treatment has significantly improved the prognosis of affected teeth. However, the primary objective in many cases is to buy time, as late complications may compromise the tooth's retention. This case report presents the long-term follow-up of an 18-year-old patient who sustained lateral luxation of Tooth 1.1 and a horizontal fracture of Tooth 2.1 due to trauma. Initial treatment included repositioning and splinting, followed by root canal therapy. Over 17 years, both teeth remained functional, although complications arose later, leading to their eventual extraction and replacement with implants. This case highlights the benefits of maintaining natural teeth in young patients and discusses potential complications with implant placement. The importance of regular follow-up and early intervention in trauma cases is emphasized, as well as the need to inform patients about the risks and benefits of different treatment approaches. The conservative management of dental trauma, especially in young patients, can effectively delay the need for implants and preserve natural teeth. Regular follow-up and early intervention are crucial to prevent complications and maintain long-term esthetic and functional outcomes.

## 1. Introduction

The protocolization of dental trauma treatment has led to significant improvements in the prognosis of affected teeth [1–6]. However, it is important to recognize that the primary goal in many cases is to buy time, as late complications may eventually compromise its retention in the dental arch [1, 4]. Horizontal fracture and lateral luxation occur predominantly in the anterior region, mainly in young male patients, resulting from car accidents, sports injuries, or physical altercations [7]. The loss of teeth in the upper esthetic area always poses a significant challenge for clinicians, particularly in young patients [8, 9]. Vertical craniofacial growth can continue until around the age of 25, and even after growth is completed, the alveolar process continues to undergo constant remodeling, particularly in the anterosuperior zone, where this growth is most pronounced [10, 11]. Teeth, thanks to the periodontal ligament, adapt to this growth, while implants remain fixed in their original position, potentially resulting in infraocclusion compared to the adjacent natural teeth [12, 13]. This infraocclusion varies from patient to patient, but it can represent a major esthetic concern [13]. Therefore, while a tooth is always preferable to an implant, this is especially true for young patients. In cases of trauma to these teeth, efforts must be made to preserve their presence in the mouth, informing the patient of the potential for short-, medium-, and long-term complications, while emphasizing the many advantages of attempting to retain the tooth [8, 14, 15].

## 2. Clinical Case

**Figure 1:**
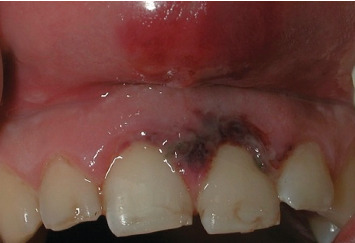
The patient presented with a lateral luxation of Tooth 1.1. Tooth 2.1 exhibited mobility and appeared extruded.

**Figure 2:**
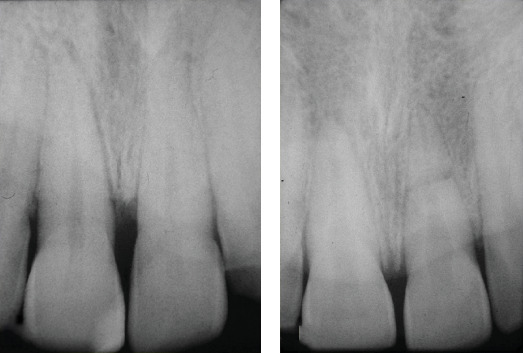
(a) In the initial periapical radiograph, no signs of fracture were observed. (b) However, after changing the orientation of the x-ray beam, the presence of a horizontal fracture became evident.

**Figure 3:**
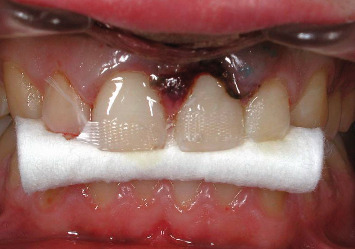
The teeth were repositioned and splinted. Following the current guidelines at that time for horizontal fractures, a rigid splint was used.

The case of an 18-year-old male patient, previously published, who suffered trauma while playing a contact sport, resulting in lateral luxation of Tooth 1.1 and a horizontal fracture of Tooth 2.1, is revisited (Figures 1 and 2). The patient provided written informed consent for the publication of this case report and accompanying images. According to the Eden Baysal index, the diagnosis for the affected teeth would have been (11)00Lm+ and (21)02Nm− [16]. Initially, Tooth 1.1 was repositioned, and Tooth 2.1 was splinted (Figure 3). At the 4-week follow-up, root canal treatment was performed on Tooth 1.1. Revascularization of the coronal fragment in Tooth 2.1 did not occur, and no union of the two fragments was achieved. Consequently, the tooth was instrumented up to the fracture line, a 6 mm mineral trioxide aggregate (MTA) plug was placed, and the remaining root canal was filled with gutta-percha, sealing the access with composite resin [17].

**Figure 4:**
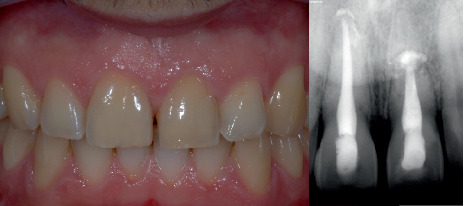
At the 10-year follow-up, neither signs nor symptoms of pathology are observed in Teeth 1.1 and 2.1.

**Figure 5:**
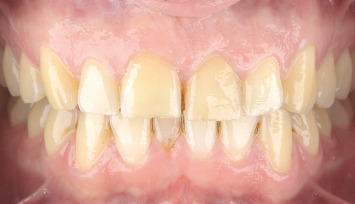
Photograph from the 17-year follow-up visit showing no signs of pathology in Teeth 1.1 and 2.1.

**Figure 6:**
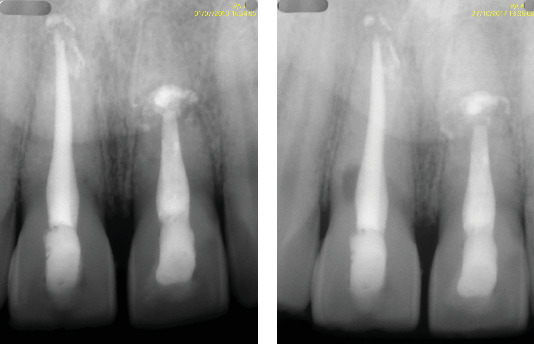
(a) The 10-year follow-up periapical radiograph shows no signs of pathology, (b) whereas the radiograph taken at 17 years shows evidence of resorption on the distal surface of the root.

**Figure 7:**
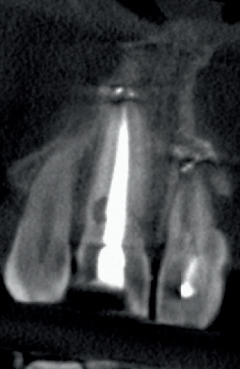
The CBCT allows for a more precise delineation of the lesion's extent at 17 years.

**Figure 8:**
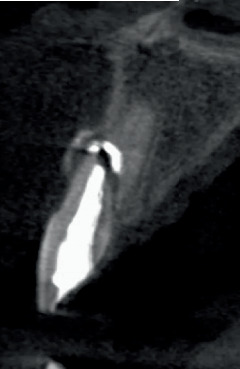
The sagittal CBCT section at 17 years of Tooth 2.1 shows displacement of the coronal fragment and the presence of a radiolucent area between the two tooth fragments.

**Figure 9:**
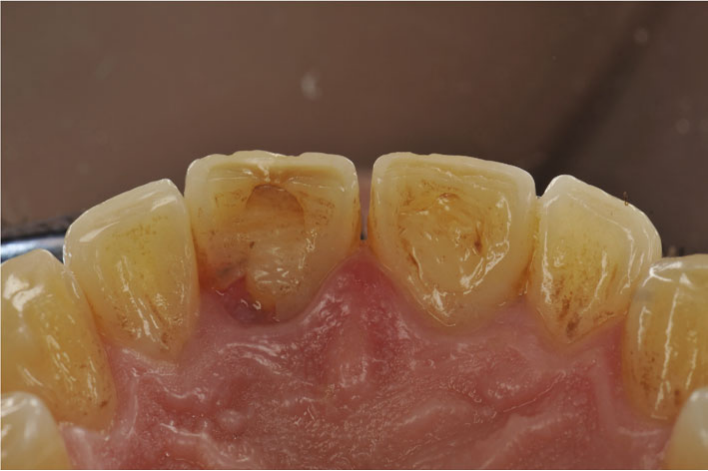
Upon inspection at 20 years, a defect was observed on the palatal surface of Tooth 1.1.

**Figure 10:**
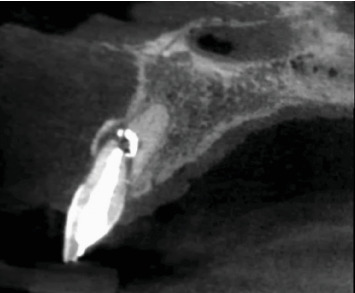
The sagittal CBCT section at 20 years of Tooth 2.1 shows greater displacement of the coronal fragment. The radiolucent area between the fragments has increased, reducing support for the coronal fragment.

**Figure 11:**
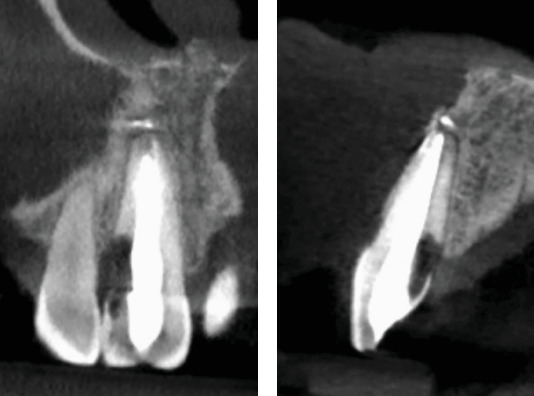
The (a) axial and (b) sagittal CBCT sections at 20 years allow us to visualize the extent of the lesion.

The patient attended regular follow-up visits and, after 10 years, exhibited excellent health, with neither signs nor symptoms of pathology. Tooth mobility and periodontal probing were within normal limits, and radiographic examination revealed no abnormalities in the surrounding tissues (Figure 4). There was no evidence of soft tissue alterations [17]. However, at the 17-year follow-up, although everything appeared normal upon inspection (Figure 5), the periapical radiograph (Carestream 6100) showed signs of resorption in the distal area of Tooth 1.1. (Figure 6). A cone beam computed tomography (CBCT) scan (Carestream 8100) revealed that the lesion was contained, and retreatment was suggested to limit its progression (Figure 7). Additionally, the coronal fragment of Tooth 2.1 had migrated relative to the apical fragment, creating a radiolucent area between the two parts, worsening the tooth's prognosis (Figure 8). Despite the recommendation, the patient did not return to the clinic until 3 years later, presenting with Grade 2 mobility in Tooth 2.1. Upon inspection, a defect was observed on the palatal surface of Tooth 1.1 (Figure 9). A new CBCT scan revealed an increase in the radiolucent area between the fragments of Tooth 2.1, along with further coronal displacement (Figure 10). It also showed that the lesion in Tooth 1.1 had progressed to an untreatable stage (Figure 11).

**Figure 12:**
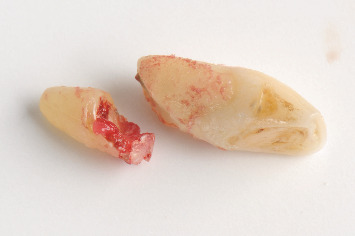
Upon extraction of Tooth 2.1, a poor crown-to-root ratio was observed, along with the presence of inflammatory tissue over the apical fragment.

**Figure 13:**
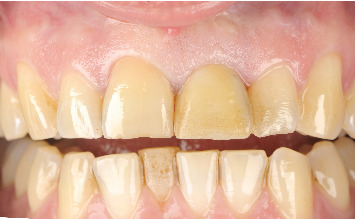
Three months after placing the implant, connective tissue graft, and provisional crown in Position 2.1, adequate healing and maturation of the soft tissues, as well as correct osseointegration of the implant, were confirmed.

**Figure 14:**
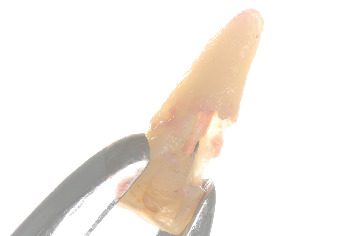
Upon extracting Tooth 1.1, the extent of the lesion, previously diagnosed with CBCT, was observed.

**Figure 15:**
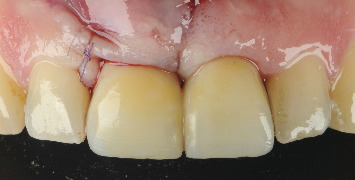
After placing the implant in Position 1.1, a connective tissue graft was performed. Due to insufficient primary stability of the implant, a provisional bridge was placed, using Tooth 2.1 as the abutment and a pontic in Position 1.1.

**Figure 16:**
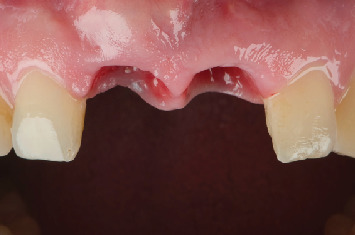
Gingival condition after contouring the emergence profile with provisional restorations.

**Figure 17:**
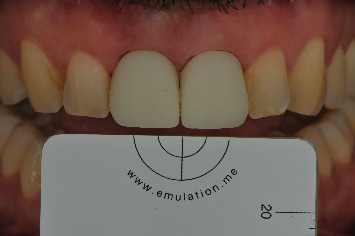
The shade was selected using the eLAB protocol.

**Figure 18:**
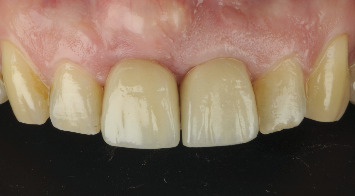
At the 3-year follow-up, although the patient is satisfied with the result, the implant-supported teeth do not fully replicate the appearance of a natural tooth. The interincisal embrasure could not be completely closed, but it is important to remember that a diastema originally existed between the natural teeth.

**Figure 19:**
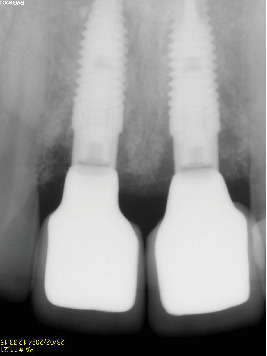
The 3-year follow-up radiograph shows no bone loss around the implants.

Following a joint assessment by the restorative dentist, endodontist, and periodontist, the decision was made to place two immediate postextraction implants in Positions 1.1 and 2.1. To minimize bone loss, a two-stage approach was chosen for implant placement. First, Tooth 2.1 was extracted (Figure 12). After the extraction, the alveolus was curetted, and an implant was placed using low-speed drilling (70 rpm) to minimize bone damage and collect autogenous bone from the drilling site [18]. The bone collected was mixed 50% with bovine xenograft and kept in saline solution. Following the placement of the implant, the gap between the implant and the vestibular bone was filled with the biomaterial and bone mixture. The implant achieved an insertion torque of 45 N/cm, allowing for immediate loading. A 3-mm-high abutment was placed, and an immediate provisional poly methyl methacrylate (PMMA) crown was milled chairside. While the provisional crown was fabricated and cemented on the titanium base, a soft tissue graft was harvested from the posterior palate and placed. After 3 months, once osseointegration of the implant in 2.1 was confirmed (Figure 13), Tooth 1.1 was extracted (Figure 14), and an immediate implant was placed following the same protocol as the implant in Position 2.1. As sufficient primary stability was not achieved, a provisional restoration was placed on 2.1 with 1.1 in extension (Figure 15). After 3 months, a 3-mm abutment was placed on the implant in Position 1.1, and two PMMA crowns were screwed to continue shaping the soft tissues. After another 3 months, with the emergence profiles deemed adequate (Figure 16), impressions were taken using an intraoral scanner. Shade matching was performed following the eLAB protocol (Figure 17) [19]. The provisional crowns were reinserted, and the patient returned after 15 days to replace the temporary crowns with the final restorations. At the 3-year follow-up, the patient is satisfied with the esthetic outcome (Figure 18), and the periapical radiograph shows that the implants are well integrated with no vertical hard tissue loss (Figure 19).

## 3. Discussion

The treatment of traumatized teeth in the anterior region, with the aim of preserving them, remains the most favorable therapeutic option, particularly in young patients. It is essential to follow the latest protocols based on scientific evidence and expert consensus [2, 6, 20]. However, it is equally important to be aware of potential short-, medium-, and long-term complications, which should be clearly communicated to the patient. The case presented, initially reported as a success at the 10-year follow-up [17], is not viewed as a failure but as an opportunity for reflection.

The prognosis for tooth loss 10 years after a midroot fracture with a closed apex is approximately 55%, although the limited number of cases in the literature calls for cautious interpretation. A poor crown-to-root ratio [21] may have contributed to the tooth's eventual loss and movement, but the presence of a radiolucent area should prompt consideration of other potential causes. The use of MTA in apexification procedures has been shown to promote effective regeneration of periapical tissues, with the potential for new cementum formation [22]. The presence of a radiolucent area in the interdental zone should raise suspicion of a possible inflammatory lesion, likely related to inadequate apical sealing of the MTA plug. This sealing defect may have been present from the initial placement or developed later, without ruling out the possibility of a fissure. We also cannot exclude the possibility that this inflammatory process originated from the apical fragment. Given that this radiolucent lesion is not visible in the periapical radiograph but is detected on the CBCT, it is advisable to consider using CBCT during follow-up evaluations for patients with these types of lesions. When considering the possibility of performing a CBCT during follow-up evaluations, the risk–benefit ratio must be carefully assessed, always bearing in mind the ALARA principle [23, 24].

While the risk of inflammatory root resorption is generally low following lateral luxation when root canal treatment is completed, its occurrence after 17 years of follow-up is not unexpected. When reviewing the prognosis of this type of pathology, we observe a growing increase in the percentage of teeth with infection-related resorption, exceeding 40% at 7.5 years. There is no information in the literature beyond 8 years, but it is reasonable to assume that the percentage of infection-related resorption will continue to rise. This would explain our findings and also suggest the importance of persisting with annual follow-ups. This approach may allow treatment before the lesion requires tooth extraction. This highlights the importance of routine checkups to identify complications, with periapical radiographs being a minimum requirement to detect issues as early as possible. Regular monitoring is vital, with early intervention potentially delaying or preventing the need for extraction and adjacent implant placement. Had intervention occurred 3 years earlier, it might have been possible to extend the life of the natural teeth and avoid immediate implants. Although guidelines recommend annual follow-ups for the first 5 years, we advocate for indefinite follow-up, given the long-term risks. Possible contributing factors for the observed resorption could include the delayed root canal treatment (4 weeks posttrauma) or latent damage to the periodontal ligament, though this remains speculative.

When contemplating the treatment of cases similar to the one presented, it is crucial to assess the prognosis of potential alternative treatments. It must also be remembered that implant treatments carry their own set of risks. Moderate to severe peri-implantitis has been reported in up to 14.5% of cases at 9 years [25], alongside other mechanical complications [26]. Furthermore, the esthetic and functional prognosis of two implants placed in the premaxilla at 18 years of age is suboptimal [8]. There is a significant risk of apparent intrusion, due to the coronal migration of adjacent teeth, as well as peri-implant tissue volume loss [27]. By delaying implant placement for nearly 20 years while preserving the hard and soft tissues in a healthy state, the patient's medium- and long-term prognosis was substantially improved.

Finally, it is crucial to stress that, after implants are placed, regular follow-ups and diligent periodontal maintenance are essential. Patients should also be thoroughly informed about the potential for short-, medium-, and long-term complications related to implants.

## 4. Conclusions

The conservative management of dental trauma, especially in young patients, can effectively delay the need for implants and preserve natural teeth. Regular follow-up and early intervention are crucial to prevent complications and maintain long-term esthetic and functional outcomes.

## Data Availability

Data sharing is not applicable to this article as no new data were created or analyzed in this study.
